# Neurobiology of Depression: Chronic Stress Alters the Glutamatergic System in the Brain—Focusing on AMPA Receptor

**DOI:** 10.3390/biomedicines10051005

**Published:** 2022-04-27

**Authors:** Ming Tatt Lee, Wei-Hao Peng, Hung-Wei Kan, Cheng-Chun Wu, Deng-Wu Wang, Yu-Cheng Ho

**Affiliations:** 1 Faculty of Pharmaceutical Sciences, UCSI University, Cheras, Kuala Lumpur 56000, Malaysia; mingtatt7286@outlook.com; 2 School of Medicine for International Students, College of Medicine, I-Shou University, Kaohsiung City 82445, Taiwan; pengweihao@isu.edu.tw (W.-H.P.); kanhw0302@isu.edu.tw (H.-W.K.); 3 School of Medicine, College of Medicine, I-Shou University, Kaohsiung City 82445, Taiwan; chengchunwu@isu.edu.tw (C.-C.W.); ed109635@edah.org.tw (D.-W.W.); 4 Department of Psychiatry, E-Da Hospital, Kaohsiung City 82445, Taiwan

**Keywords:** glutamate, AMPA, NMDA, depression, chronic stress, prefrontal cortex, hippocampus, amygdala, nucleus accumbens, periaqueductal gray

## Abstract

Major depressive disorder (MDD) is a common neuropsychiatric disorder affecting the mood and mental well-being. Its pathophysiology remains elusive due to the complexity and heterogeneity of this disorder that affects millions of individuals worldwide. Chronic stress is frequently cited as the one of the risk factors for MDD. To date, the conventional monoaminergic theory (serotonin, norepinephrine, and/or dopamine dysregulation) has received the most attention in the treatment of MDD, and all available classes of antidepressants target these monoaminergic systems. However, the contributions of other neurotransmitter systems in MDD have been widely reported. Emerging preclinical and clinical findings reveal that maladaptive glutamatergic neurotransmission might underlie the pathophysiology of MDD, thus revealing its critical role in the neurobiology of MDD and as the therapeutic target. Aiming beyond the monoaminergic hypothesis, studies of the neurobiological mechanisms underlying the stress-induced impairment of AMPA (a-amino-3-hydroxy-5-methyl-4-isoxazole propionic acid)-glutamatergic neurotransmission in the brain could provide novel insights for the development of a new generation of antidepressants without the detrimental side effects. Here, the authors reviewed the recent literature focusing on the role of AMPA-glutamatergic neurotransmission in stress-induced maladaptive responses in emotional and mood-associated brain regions, including the hippocampus, amygdala, prefrontal cortex, nucleus accumbens and periaqueductal gray.

## 1. Introduction

Major depressive disorder (MDD) is a heterogeneous neuropsychological disorder characterized by a combination of symptoms that negatively impact the productivity and well-being of inflicted patients, including impairments in cognition, emotional regulation, memory, motor function, motivation, and possible suicidal ideation. Approximately 280 million people in the world are affected by this psychiatric disorder, and it is regarded as one of the leading causes of morbidity and disability worldwide [1]. An episode of major depression may occur once in a person’s lifetime, but it is more likely to relapse throughout a person’s life [2]. Although several pharmaceutical strategies have been proven in clinical settings, more than two-thirds of patients with MDD do not achieve stable remission of symptoms, despite currently available medication [3]. Furthermore, the current antidepressant drugs targeting the monoamine systems (norepinephrine, dopamine, and/or serotonin) require a long duration (4–8 weeks) to take effect [4]. The severe adverse effects associated with the pharmacotherapy, including cardiac toxicity, hyperpiesia, sexual dysfunction, body weight gain, and sleep disorders, often hamper patients’ adherence [5,6]. The current pharmacotherapy for MDD remains unsatisfactory, which may be attributed to the lack of understanding of the neurobiological mechanisms underlying this pathological condition.

MDD is a debilitating disorder with multiple potential etiologies, including environmental and genetical influences [7]. Although several hypotheses have been suggested to explain the pathophysiological mechanisms of MDD, the contours of the disorder are still unclear. Among these hypotheses, the dysregulation of neurotransmitters has received the most attention in the neurobiological mechanisms of MDD, i.e., the monoaminergic hypothesis involving serotonin, norepinephrine, and/or dopamine. Clinically, most antidepressants target this system as a therapeutic goal, including tricyclic antidepressants, selective serotonin reuptake inhibitors (SSRIs), serotonin, and norepinephrine reuptake inhibitors (SNRIs), to restore the levels of these monoamines [8].

A growing consensus posits that altered monoaminergic transmission is insufficient to explain the etiology of depressive disorders [9,10,11]. Hypothalamic–pituitary–adrenal (HPA) axis dysregulation has been implicated in the pathogenesis of MDD, as the hyperactivity state of the HPA axis is one of the most consistent findings in the neuroendocrinology of depression [12,13] and as the HPA axis is dysregulated by stress and trauma, both of which are known precipitants of MDD [14]. Furthermore, data from rodents showed that central administration of corticosterone, a stress-related hormone, can produce depression-like behaviors [15]. The plasma levels of corticosterone, a stress hormone, have been widely used as biomarkers of stress [16], depression [17] and anxiety [18] in mice, and such a phenomenon was also observed in humans [19] in the form of plasma cortisol. Chronic stress is reported to induce neuropsychological disorders, including depression and anxiety, via the hyperactivity of the hypothalamic–pituitary–adrenal (HPA) axis [20,21].

In addition to the monoaminergic system, a growing body of evidence has revealed that the glutamatergic system plays a critical role in the pathophysiological mechanisms of MDD [1,22,23]. Several postmortem studies have indicated that glutamate receptor subunits, including the GluR1 subunit and AMPA binding, were reduced in patients with major depression [22,23,24,25]. Beyond the conventional monoaminergic theory, these findings provided an alternative concept in the possible pathological mechanisms of MDD and the different approach in the development of novel antidepressants.

A literature search was conducted in March 2022 in PubMed for literature published from January 2000 onward using the following terms: “Depression” OR “Depressive” AND “Glutamatergic transmission” AND “Brain” AND “AMPA”. This manuscript is an original review highlighting the central role played by the glutamatergic system in mediating stress-associated maladaptive pathological responses in the central nervous system, especially in emotional-related brain regions. A central, recurrent theme throughout this review is a comprehensive summary of the effects of stress exposure on glutamatergic synapses. Indeed, several reports show a thorough analysis of the correlation between stress and depression with altered brain volume, synaptic remodeling, and maladaptive connectivity between brain nuclei. As a result, we go over the evidence for neuroplastic changes at the synaptic level in depression, focusing on glutamatergic transmission in depression. This review sought to discuss the role of glutamatergic transmission, with emphasis on the AMPA receptor, in the development of MDD. The synaptic changes and functional plasticity of AMPA-glutamatergic transmission in several brain regions related to MDD were discussed.

## 2. A Brief Overview of Glutamate Neurotransmission in the Brain

**Figure 1:**
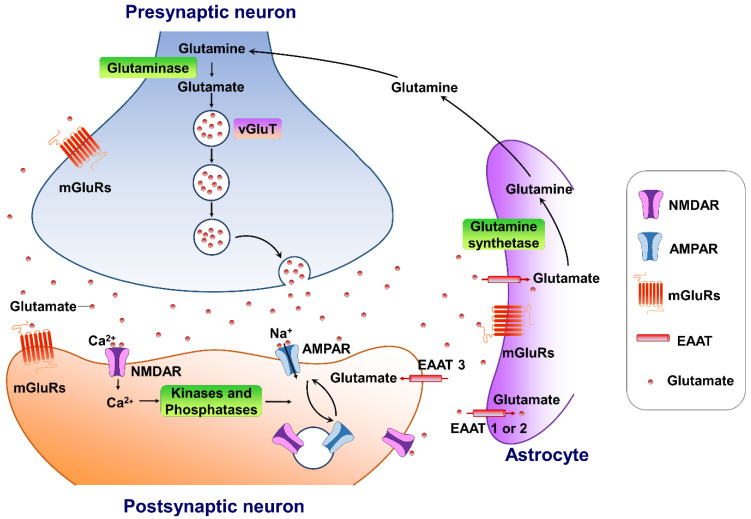
**The tripartite glutamate synapses.** Most glutamate molecules are cleared from the synaptic cleft through the excitatory amino-acid transporter (EAAT 1/2) located on the astrocytes. Within the astrocyte, glutamine synthetase converts glutamate to glutamine, and the glutamine is subsequently released from the astrocyte and taken up by neighboring neurons to complete the glutamate–glutamine cycle in the brain. Neuronal glutamate is synthesized *de novo* from glutamine originating from nearby astrocytes. Glutamate is then loaded into synaptic vesicles by vesicular glutamate transporters (VGLUTs). Upon being triggered by an action potential, glutamate will be rapidly released into the synaptic cleft. Here, glutamate binds to either ionotropic glutamate receptors (AMPA receptors and NMDA receptors) and/or metabotropic glutamate receptors (mGluRs) on the membranes of both postsynaptic and presynaptic neurons and astrocytes. Upon activation, these glutamate receptors initiate various cellular responses, including depolarization of membrane potential, activation of intracellular signaling, regulation of protein synthesis, and/or gene expression. Surface expression and functional alteration of AMPARs and NMDARs are dynamically mediated by protein synthesis and degradation. The receptors traffic between the postsynaptic membrane and endosomes to maintain the dynamic adaptation/alteration of physiological/pathological responses.

Glutamate is the major excitatory neurotransmitter in the central nervous system. It plays an important role in several physiological functions. A typical glutamate synapse is composed of three distinct cell types: an astrocyte, a presynaptic neuron, and a postsynaptic neuron [26]. In the central nervous system, glutamine synthesis from glutamate and ammonia occurs exclusively in the astrocytes. Then, glutaminase, a precursor for the synthesis of the neurotransmitter glutamate, hydrolyses glutamine to glutamate and ammonia within neurons, completing the glutamate–glutamine cycle in the brain (Figure 1). Glutamate is then packaged into vesicles in the presynaptic neuron by three types of vesicular glutamate transporters (VGLUT1, VGLUT2 and VGLUT3) and released into the synapse from the presynaptic terminals [27], in an activity-dependent manner. The released glutamate in the synaptic cleft can engage both ionotropic and metabotropic glutamate receptors, located in the presynaptic and postsynaptic neurons, as well as on astrocytes [28]. The ionotropic subtypes of glutamate receptors, including the AMPA, *N*-methyl-D-aspartate (NMDA), and kainate receptors, control fast excitatory neurotransmission, whereas the family of eight metabotropic glutamate receptors (mGluR1-8) are located in presynaptic areas, postsynaptic areas, and extra-synaptically throughout the central nervous system [29]. The concentrations of synaptic glutamate are regulated by glutamate transporters localized in glial cells and neurons due to the fact that there is no process by which glutamate is metabolized in neurons.

## 3. Synaptic Plasticity of Chronic Stressor Priming

The synaptic plasticity of chronic stressor priming proposes that sustained stressors cause widespread neuronal remodeling consistent with both diminished and increased synaptic connectivity, depending on the brain nuclei. Structural and functional changes in several brain areas led by chronic stress have been widely explored. The effect of chronic stress in reducing synaptic strength and connectivity were mostly investigated in the prefrontal cortex (PFC), hippocampus and the periaqueductal gray. On the other hand, chronic stress has been reported to increase synaptic connectivity, characterized by increased dendritic length, in the nucleus accumbens (NAc) and certain nuclei of the amygdala. These observations reveal that stress-induced depression is caused by a disruption in homeostatic mechanisms controlling synaptic strength. This breakdown of synaptic homeostasis leads to the destabilization and loss/gain of synaptic connections in mood and emotion-associated circuitry. In the following sections, we mainly focus on the neuroplastic changes of glutamatergic transmission in specific brain areas of mood-related neural circuity.

### 3.1. Hippocampus

Convergent lines of research demonstrate that the hippocampus, a brain area involved in learning and in the consolidation of explicit memories, is susceptible to chronic stress and plays an important role in the pathophysiology of MDD [30]. The hippocampus is vulnerable to atrophy in patients with MDD [31], as reduced volume of the hippocampus may be resultant of neuronal remodeling in MDD [32]. In preclinical models of chronic stress-induced depression models, hippocampal neurogenesis [33] and dendritic spine density/complexity [34,35] were markedly reduced, and these phenomena are related to MDD in clinical settings [36,37]. It is noteworthy that hippocampal chronic stress-induced depression-like responses were negatively correlated with the levels of dendritic complexity, dendritic spine densities, and synaptosomal AMPA receptors [38]. These structural and functional deficits may further contribute to stress dysregulation as they affect the hippocampus’s inhibitory signal to the HPA axis [39,40,41].

In addition to structural remodeling, chronic stress is known to disrupt functional connections within the neural network. However, neuronal adaptation is quite different between variable stress paradigms. Acute stress potentiated the AMPA receptor-mediated glutamatergic transmission in the hippocampus [42], while chronic stress impaired the AMPA receptor-dependent synaptic transmission in the hippocampus [43]. Chronic stress sustainably elevated the level of corticosterone, which is the key factor of stress-induced depression-like behavioral changes and synaptic dysfunction, suggesting the involvement of HPA activation. A previous study demonstrated that chronic stress caused a reduction in AMPA receptor-mediated synaptic strength at temporoammonic synapses [43]. In addition, repeated administration of exogenous corticosterone produced impaired synaptic transmission characterized by reduced AMPA receptor-mediated excitation at temporoammonic-CA1 synapses and decreased AMPA receptor subunit 1 protein expression [44]. In contrast to the disruption of AMPA receptor-mediated transmission produced by repeated corticosterone treatment, acute corticosterone enhances transmission at temporoammonic-CA1 synapses [44,45]. These phenomena are consistent with previous findings on the differential effects of acute vs. chronic stress on the AMPA receptor-mediated glutamatergic transmission in the hippocampus [42,43].

In addition to directly interfering with AMPA-glutamatergic transmission, stress is also reported to be capable of triggering the inflammatory processes regulating AMPA receptor-mediated transmission [46]. Chronic stress can induce caspase-1/IL-1b generation, leading to AMPA receptor internalization in the hippocampal neurons, which disrupted synaptic glutamatergic transmission, eventually causing depression-like behavior [46]. Therefore, restoring excitatory strength, especially AMPA receptor-mediated fast synaptic transmission, at stress-sensitive synapses in the hippocampus seems to be a critical mechanism for maintaining normal cognitive and emotional behavior.

### 3.2. Amygdala

The amygdala is a brain region important for the integration of emotional processes and emotion-related memories, which receives input from divergent brain regions involved in emotion, such as the PFC, thalamus, hippocampus, raphe, and VTA [47]. The basolateral amygdala (BLA), a subregion of the amygdala, plays a crucial role in stress-induced emotional disorders, and has been widely implicated in the pathophysiology of MDD. In the BLA, chronic restraint stress in mice upregulated A-kinase anchoring proteins, leading to the insertion of GluA1-containing AMPA receptors into the postsynaptic membrane [48]. The increased AMPA receptor-mediated excitatory synaptic transmission eventually resulted in the manifestation of depression-like behaviors in mice. Moreover, another report revealed that enhanced synaptic recruitment of AMPA receptor via protein kinase A-dependent mechanisms in the BLA was essential for chronic restraint stress-evoked depression-like behavior [49].

Morphologically, repeated stress can remodel GluR1-containing AMPA receptors from dendritic stores into the spine region and drive an increase in excitatory transmission in vivo in BLA neurons after chronic stress [50,51]. Furthermore, another study revealed that chronic stress can induce the insertion of calcium-permeable subunits into the AMPA receptors of the BLA neurons, thus increasing the efficiency of AMPA-glutamatergic transmission and modulating emotional behaviors in mice [52]. This evidence could, at least in part, explain the enhanced BLA activity under exposure to chronic stress [53].

### 3.3. Prefrontal Cortex

The PFC, a cortical region located in the anterior part of the frontal lobe, is the main brain region governing the higher cognitive functions and emotional processes. The PFC projects long-range fibers to diverse subcortical autonomic, limbic, and motor regions. On the other hand, the PFC receives afferents from various brain regions involved in sensory and emotional integration, including the hippocampus, thalamus, basolateral amygdala (BLA), etc. These anatomical connections signify the wide range of physiological functions controlled by the PFC. A growing body of evidence demonstrates that medial PFC (mPFC) dysfunction plays an important role in the pathophysiology of MDD [54]. Previous human transcriptomic studies of PFC suggested that MDD is associated with aberrant synaptic transmission in the PFC, which affects the glutamatergic systems [55,56]. Given that the PFC is known to be susceptible to chronic stress, which underlies the pathogenesis of depression [57], the AMPA-glutamatergic alteration in PFC following chronic stress may be another interesting point of review.

Similar to other brain regions, the variable duration of stress exposure can cause varied effects in the AMPA-glutamatergic system in the PFC. A previous study indicated that acute stress elicited the enhancement of AMPA receptor-mediated glutamatergic transmission and contributed to increased activity of the PFC circuits, hence leading to an improvement in working memory performance in rats [58]. In contrast, prolonged stress disrupts AMPA receptor-mediated glutamatergic transmission, leading to aggression and violence, while chemogenetic activation of PFC pyramidal neurons restores AMPA receptor-mediated glutamatergic transmission, leading to the alleviation of behavioral abnormalities [59]. In addition, chronic restraint stress-induced selective loss of p11, a multifunctional protein in the brain bound to 5-HT receptors, resulting in diminished AMPA receptor-mediated synaptic transmission in the PFC, contributing to depression-like behavior [60]. Moreover, chronic stress impairs synaptic transmission and degrades AMPA receptors in the PFC, leading to depression-like behavior via epigenetic mechanisms [61]. These reports indicated that prolonged exposure to stressor causes hypoconnectivity of the neuronal network in the PFC, as evidenced by decreased AMPA receptor function and expression, resulting in depression-like behavior. The potential role of the AMPA-glutamatergic system in PFC can be further supported by an in vivo study, which demonstrated that activation of mPFC AMPA receptors can alleviate chronic, mild stress-induced depressive-like behaviors in rats [62].

### 3.4. Nucleus Accumbens

The NAc, a key target brain area involved in reward processing and substance dependence, also receives a dense glutamatergic innervation from the PFC, amygdala, and hippocampus [63,64], thereby influencing the motivation to seek rewarding stimuli [65]. Anhedonia, manifested as reduced motivation to seek pleasure activities, is one of the core symptoms of MDD that is partly influenced by NAc [64,66]. In fact, lower resting-state functional connectivity [67] in NAc was observed in brain imaging studies of MDD patients with anhedonia. Furthermore, in rats subjected to chronic mild stress, hypertrophy of the GABAergic medium spiny neurons (MSN) was observed, correlating with anhedonic behavior and reversible by antidepressants, fluoxetine and imipramine [66].

In terms of AMPA-glutamatergic transmission in the NAc, a previous report demonstrated that chronic restraint stress reduces AMPA receptor-mediated synaptic transmission in D_1_ receptor-expressing MSNs in the NAc [68]. The diminished glutamatergic transmission is displayed in parallel with depression-like behavior, including anhedonia and despaired behavior. Moreover, the same study showed that the prevention of stress-induced reduction in AMPA receptor-mediated excitatory transmission abolished anhedonia in rodents [68]. As such, it is evident that chronic stress-evoked decrement of AMPA receptor-mediated excitatory transmission is necessary for the manifestation of depression-associated symptoms, specifically anhedonia, highlighting the critical role of AMPA receptors in the reward circuits for the genesis of anhedonia.

### 3.5. Periaqueductal Gray

Periaqueductal gray (PAG) is a brain nucleus that regulates pain-associated responses, autonomic function, analgesia, and psychological stress-related behaviors [69,70,71,72]. The role of PAG has been widely explored in its regulatory action on nociceptive transmission [70,71,73,74,75,76]. Although it is not part of the limbic system, growing evidence strongly suggests that the PAG is also a key player in regulating chronic stress-induced depression-like behaviors [72,77,78,79,80,81]. Repeated foot shock-induced stress produces learned helplessness-related depression-like behavior including despaired behavior and anhedonia [78,79]. This depression-like behavior is parallel with decreased AMPA receptor-mediated glutamatergic transmission in the ventrolateral PAG (vlPAG). The diminished glutamatergic transmission originated from the reduced presynaptic glutamate release and decreased postsynaptic GluR1-containing AMPA receptor function. In addition, exogeneous administration of a glucocorticoid receptor agonist mimicked repeated foot shock stress-provoked impairment of glutamatergic transmission in the vlPAG. This hypofunction of glutamatergic transmission in the vlPAG contributes to chronic stress-induced depression-like behavior through glucocorticoid receptor-dependent mechanisms. Moreover, pharmacological activation of the vlPAG neurons by microinjection of ketamine metabolite, (*2R,6R*)-hydroxynorketamine (HKN) restored the learned helplessness-induced depression-like behavior as well as learned helplessness-evoked decreased glutamatergic transmission in the vlPAG [78]. Mechanistically, (*2R,6R*)-HKN alleviated learned helplessness-induced depression-like behavior through its actions in the vlPAG by enhancing presynaptic glutamate release and increasing postsynaptic AMPA receptor function [78]. Furthermore, (*2R,6R*)-HKN directly increased glutamate release presynaptically, and postsynaptic AMPA receptor function are sufficient to trigger depolarization in vlPAG neurons, resulting in elevated vlPAG neuronal activity [72,78]. These may imply that the reversal of impaired excitatory synaptic transmission in the vlPAG can alleviate chronic stress-elicited depression-like behavior. GLYX-13, a novel glutamatergic compound that acts as an NMDA receptor positive allosteric modulator, has been evidenced as a rapid-acting and long-lasting antidepressant in preclinical studies [77,82,83,84]. Intra-vlPAG microinjection of GLYX-13 produced rapid-onset antidepressant effects by which GLYX-13 enhanced AMPA receptor-mediated glutamatergic transmission in the vlPAG via the BDNF-TrkB-mTORC1 cascades, leading to a sustained alleviation of chronic stress-elicited depression-like behavior.

**Figure 2:**
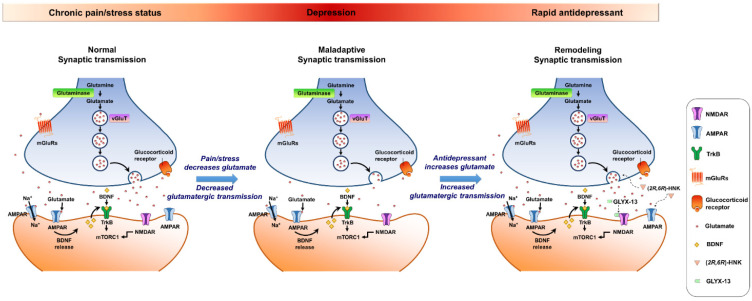
A proposed model for the maladaptation of synapses caused by chronic stress and proposed mechanisms of action of glutamatergic agents in the midbrain ventrolateral periaqueductal gray (vlPAG). Synaptic strength in the vlPAG is maintained at normal neural activity, but is diminished by chronic stress exposure, which depresses glutamate release presynaptically and AMPAR expression postsynaptically. (*2R,6R*)-hydroxynorketamine (HNK), the metabolite of ketamine that exhibits a fast-acting antidepressant effect, can rapidly reverse the effects of chronic stress by releasing a large amount of glutamate that leads to the insertion of AMPARs onto the postsynaptic membrane. GLYX-13, a partial agonist at NMDARs, is hypothesized to initiate mammalian targets of rapamycin complex 1 (mTORC1) and subsequently induce protein synthesis. GLYX-13 requires AMPARs activation and triggers activity-dependent brain-derived neurotrophic factor (BDNF) release. AMPARs activation increases BDNF release, engages the tropomyosin receptor kinase B (TrkB) receptor, and eventually triggers protein synthesis by activating the mTORC1 cascades. It is noteworthy that similar maladaptive synaptic transmission was observed in preclinical models of chronic pain, another risk factor for depression.

It has been previously reported that chronic pain caused by sustained tissue injury is a type of stressor that weakens glutamatergic transmission in the vlPAG [71,73], but it is unclear whether psychological stress can exert similar changes in glutamatergic transmission in the vlPAG, leading to depression-like behavior. A recent study demonstrates that restraint stress causes depression-like behavior, which is in parallel with suppressed AMPA receptor-mediated synaptic transmission in the vlPAG [72]. Consistently, the reversal of depressed glutamatergic transmission in the vlPAG by microinjection of (*2R,6R*)-HKN rescued the depression-like behavior. These reports suggest that chronic stress (both physiological and psychological stressors) disrupts AMPA receptor-medicated synaptic transmission in the vlPAG contributing to depression-like behavior, whereas the restoration of diminished glutamatergic transmission leads to amelioration of depression-like behavior. The AMPA receptor-mediated glutamatergic transmission in the vlPAG might be a crucial biomarker in depression-associated emotional and reward circuits (Figure 2).

In addition to neuronal cells, astrocytes are the most numerous neuroglial cells in the central nervous system. Astrocytes release several gliotransmitters and influence neuronal activity and plasticity [85]. Restraint water immersion stress elicited astrocytic activation in the vlPAG contributing to stress-induced gastric mucosal damage [86]. Moreover, chronic restraint stress decreases glial fibrillary acidic protein and glutamate transporters in the vlPAG [87]. A glutamate transporter disruption affects glutamate content at synaptic clefts, resulting in dysregulation of glutamatergic transmission. Maladaptive excitatory transmission in the vlPAG causes dysfunction of the PAG after stress priming.

## 4. Targeting AMPA-Glutamatergic Signaling for the Development of Novel Therapeutics for Depressive Disorders

Emerging evidence of glutamatergic synaptic transmission dysfunctions demonstrates how this defect correlates with the pathogenesis of depression. MDD is attributed to a weakening of specific subsets of excitatory synapses in various brain nuclei that are important in the processing of affect and reward (Table 1). The critical action of effective antidepressants is the reversal of the diminished excitatory synaptic strength in these impaired brain areas. A candidate agent verified in preclinical models that promotes stress-sensitive excitatory synapses in the emotional and reward circuits may be an effective antidepressant. Reduced glutamate levels have been noted in several neural regions of patients with MDD [88], whereas several glutamatergic agents have been proven to effectively alleviate depressive symptoms in patients with MDD [89]. Accumulating evidence demonstrates that transcriptional regulation is involved in various levels of regulation of gene expression [90]. Chronic stress causes a loss of AMPA receptors, and GluR1 expression in the PFC is attributed to the enhancement of ubiquitin–proteasome-dependent degradation of GluR1 controlling by E3 ubiquitin ligase [91]. Transcriptional regulation of gene expression has been considered as a key player underlying the persistent impact of stress response.

**Figure 3:**
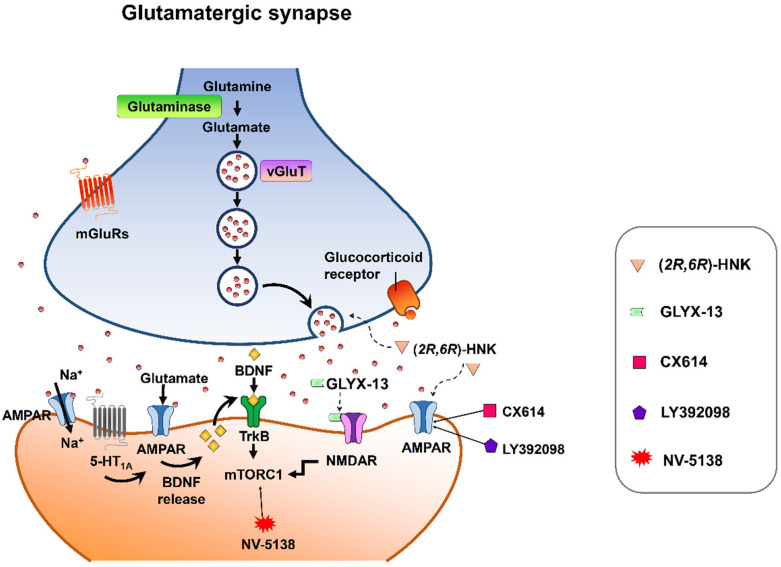
Synaptic model for the cellular target sites for different types of candidate drugs for antidepressants. (*2R,6R*)-HNK exerts increased glutamate release and AMPA receptor-mediated synaptic potentiation. GLYX-13 elicited partial activation of the NMDA receptor, hence activation of mTORC1 and thus induction of protein synthesis. CX614 and LY392098, AMPA receptor potentiators, induce antidepressant effects by enhancement of AMPA receptor function and BDNF expression. NV-5138 exerts rapid and sustained antidepressant effects through stimulating mTORC1 signaling. Activation of the 5-HT_1A_ receptor produces rapid and sustained antidepressant effects through the initiation of AMPA receptor/BDNF/mTORC1 cascades. All the candidates propose long-lasting modifications in synaptic plasticity, resulting in strengthening of glutamatergic synapses, which is necessary for antidepressant responses.

Despite mounting positive preclinical results, most AMPA receptor-targeting agents have failed to declare any relevant clinical effects in treating neuropsychiatric disorders. This discrepancy may be due to multiple factors. Nonetheless, strong preclinical evidence supports that AMPA receptors are involved in the therapeutic effects of current antidepressant drugs. Chronic restraint stress-induced depression in rodents was reversed by the metabolite of ketamine, (*2R,6R*)-HNK, via AMPA receptors [113] at the concentration that do not block NMDA receptor function [114]. On the other hand, mPFC AMPA receptor and BDNF signaling are required for the rapid and sustained antidepressant-like effects of 5-HT_1A_ receptor stimulation [115]. Interestingly, pharmacological enhancement of AMPA transmission has been reported to synergize the antidepressant potency of conventional antidepressants, e.g., fluoxetine, imipramine, and rolipram [116]. Given that AMPA-glutamatergic neurotransmission in various stress-related brain regions signifies the dysfunction of excitatory synapse, it could point to a new strategy for developing novel antidepressants or as a target for adjunct therapy alongside current antidepressants (Figure 3).

## 5. Conclusions

Chronic stress elicits intricate functional and structural alterations in various brain regions. With regard to the glutamatergic synapse, chronic stress is known to either strengthen or weaken effects that are associated with improved or impaired function, respectively, and this change may contribute to the pathophysiology of neuropsychiatric disorders. Mounting evidence reveals the critical role of homeostatic control of synaptic connections in mood-associated circuits in the etiology and treatment of depression. Further research is required to explore the effects of chronic stress and antidepressants on different brain areas and the mood-related neural circuits and to unmask the functional importance of synaptic maladaptation on behavior changes. These preclinical studies will further determine the neuronal and synaptic changes underlying depression and will contribute to the development of more efficacious, rapid-acting, sustained, and safer antidepressants. Although there is still enormous undiscovered neural maladaptation in the diseased brain, to date, these results reveal insight into the pathogenesis of depression and provide a novel strategy for refining therapeutic approaches to depressive disorders.

## Figures and Tables

**Table 1:** Effects of AMPA receptor response to different stress paradigms. ↑ = increase; ↓ = decrease; - = no change.

Classification	Type of Stress	Brain Area	Effects on AMPA Receptor	Reference
Acute stress	Restraint stress for 2 h	Hippocampus	GluA1 subunit phosphorylation ↑	[92]
		Basolateral amygdala	GluA1 subunit phosphorylation -	[92]
		Frontal cortex	GluA1 subunit phosphorylation -	[92]
	Restraint stress for 30 min	Hippocampus	GluA1 subunit phosphorylation ↑ GluA1 expression ↑	[93]
	Unsteady platform for acute stress	Hippocampus	GluA1 expression ↓	[94]
	Acute footshock stress	Prefrontal and frontal cortex	GluA1 subunit phosphorylation ↑ GluA2 subunit phosphorylation ↑	[95]
	Elevated platform stress	Amygdala	GluA1 subunit phosphorylation ↑	[96]
		mPFC	GluA1 subunit phosphorylation ↑	[96]
		Hippocampus	GluA1 subunit phosphorylation ↑	[96]
	Acute restraint stress for 1 h	Hippocampus	GluA1 expression - GluA2 expression -	[97]
	Acute restraint stress for 30 min	Hippocampus	GluA1 subunit phosphorylation ↑	[98]
	Elevated platform stress	Hippocampus	GluA2 expression ↓	[99]
	Restraint or forced swimming	Amygdala	GluA1 subunit phosphorylation ↑GluA1 expression ↑	[100]
	Acute restraint stress for 2 h	Nucleus accumbens	GluA1 expression ↑	[101]
	Unsteady platform for 30 min	mPFC	Ser831-GluA1 phosphorylation ↓ Ser880-GluA2 phosphorylation ↑	[102]
		Hippocampus	Ser831-GluA1 phosphorylation ↓	[102]
		Amygdala	Ser845-GluA1 phosphorylation ↑ Tyr876-GluA2 phosphorylation ↓ Ser880-GluA2 phosphorylation ↓	[102]
	Forced-swim stress	Prefrontal cortex	Surface GluA1 expression ↑ Surface GluA2 expression ↑	[103]
	Immobilization stress for 45 min	Hippocampus	AMPA mRNA levels -	[104]
	Acute restraint stress for 6 h	Dentate gyrus	GluR2 flip mRNA expression↑	[105]
Chronic stress	Chronic mild stress	Hippocampus	AMPA mRNA - GluA2 expression ↑	[106]
	Chronic unpredictable mild stress	Hippocampus	GluA1 expression - GluA2 expression↑ GluA3 expression ↑	[107]
	Early life Stress	Hippocampus	NMDA/AMPA ratio ↓	[108]
	Chronic unpredictable mild stress	Hippocampus	GluA1 expression ↓ GluA2 expression ↓ GluA1 subunit phosphorylation ↓	[109]
	Week chronic mild stress	Hippocampus	GluA1 expression ↓	[110]
	Chronic unpredictable stress	Hippocampus	GluA1 expression ↓	[44]
	Neonatal isolation stress	Paraventricular nucleus	AMPA binding sites ↑	[111]
	Chronic immobilized stress	Nucleus accumbens	GluA1 expression ↑	[112]
	Immobilization stress for 14 days	Hippocampus	AMPA mRNA levels -	[104]
	Chronic restraint stress for 21 days	Hippocampus CA1	GluR1 flip mRNA expression ↓	[105]

## Data Availability

Data is contained within the article.
